# Prognostic value of visual residual tumour cells (VRTC) for patients with esophageal squamous cell carcinomas after neoadjuvant therapy followed by surgery

**DOI:** 10.1186/s12885-020-07779-0

**Published:** 2021-02-03

**Authors:** Xingxing Wang, Hao Wang, Haixing Wang, Jie Huang, Xin Wang, Zhengzeng Jiang, Lijie Tan, Dongxian Jiang, Yingyong Hou

**Affiliations:** 1 Department of Pathology, Zhongshan Hospital, Fudan University, Shanghai, 200032 People’s Republic of China; 2 Department of Thoracic surgery, Zhongshan Hospital, Fudan University, Shanghai, 200032 People’s Republic of China; 3 Department of Pathology, School of Basic Medical Sciences & Zhongshan Hospital, Fudan University, Shanghai, 200032 People’s Republic of China; 4 Department of Pathology, Qingpu Branch of Zhongshan Hospital, Fudan University, Shanghai, 201700 People’s Republic of China

**Keywords:** Neoadjuvant therapy, Esophageal squamous cell carcinoma (ESCC), Visual residual tumour cells (VRTC), Tumour regression grading (TRG), Lymph node metastases (LNM)

## Abstract

**Background:**

We assessed visual residual tumour cells (VRTC) with both Becker’s tumour regression grading (TRG) system and Japanese TRG system in esophageal squamous cell carcinoma (ESCC) patients treated with neoadjuvant therapy followed by surgery.

**Methods:**

We compared Becker system and Japanese system in 175 ESCC patients treated between 2009 and 2015.

**Results:**

According to Becker system, the 5-year DFS/DSS rates were 70.0%/89.3, 53.8%/56.7, 43.0%/49.0, and 42.4%/39.1% for TRG 1a (VRTC 0), TRG 1b (1–10%), TRG 2 (11–50%), and TRG 3 (> 50%). According to Japanese system, the rates were 38.8%/34.1, 49.5%/58.7, 50.2%/49.0 and 70.0%/89.3% for Grade 0-1a (VRTC> 66.6%), Grade 1b (33.3–66.6%), Grade 2 (1–33.3%) and Grade 3 (0). TRG according to two systems significantly discriminate the patients’ prognosis. TRG according to Becker system (HR 2.662, 95% CI 1.151–6.157), and lymph node metastasis (HR 2.567, 95% CI 1.442–4.570) were independent parameters of DSS.

**Conclusions:**

Both Becker and Japanese system had their advantage in risk stratification of these ESCC patients. It was speculated that dividing 1–10% VRTC into a group might contribute to independently prognostic significance of Becker’s TRG system. Therefore, in addition to TRG of different systems, the percentage of VRTC might be recommended in the pathologic report, which could make the results more comparable among different researches, and more understandable for oncologists in the clinical practice.

## Background

Esophageal cancer (EC) is the ninth most common cancer and the sixth most common cause of cancer death globally [1]. Chinese population-based studies have shown approximately 477,900 persons receive a diagnosis of EC, and 375,000 persons died of EC in 2015, ranking it as the third most commonly diagnosed cancer and the fourth leading causes of cancer death in China [2]. Surgical resection has been the mainstay of treatment for EC. However, the majority of patients with locally advanced EC who undergo surgical resection eventually develop local recurrence or distant metastasis, and the 5-year survival rate is only 5–34% [3]. In an attempt to improve survival, many investigators around the world have assessed multidisciplinary strategies. The preoperative neoadjuvant chemotherapy (nCT) or chemoradiotherapy (nCRT) combined with surgery have gained more attention in the treatment of locally advanced EC [4, 5]. Several studies have shown that neoadjuvant therapy (nCT or nCRT) followed by surgery significantly improves disease free survival (DFS) and overall survival (OS) compared with surgery alone, making it standard therapy for locally advanced EC [6–9]. Accumulating evidence indicates that the histological evaluation of the regression response to nCT or nCRT is the most important predictor of survival [5, 10].

Tumour regression grade (TRG) system referring to the amount of therapy-induced fibrosis in relation to residual tumour after nCRT has been initially developed by Mandard and coworkers [11]. However, the reproducibility and prognostic value of this system has been challenged because of the difficulties in the assessment of the relative amount of fibrosis [12]. Then the quantitative analysis of regression response through the estimated percentage of visual residual tumour cell (VRTC) in relation to the previous tumour site has been proposed. At present, there are several classification systems (2 to 5 grades) available in the literature, with the cut-off value of 1, 10 and 50% [13–18]. Among the systems, a modification of 4-tiered Becker system not only resulted in statistically superior rates for interobserver agreement but also in achievement of a better prognostic impact in many reports [19]. Besides, TRG system with cutoff value of 1/3 and 2/3, according to Japanese Classification of Esophageal Cancer, has also been widely used in esophageal squamous cell carcinoma (ESCC) [20, 21]. The larger retrospective studies found that the Japanese system was also simple, reproducible and prognosis-associated [22, 23]. At present, there was no study to compare the application of the 2 major approaches (Becker system and Japanese system) for assessment of VRTC in ESCC after neoadjuvant therapy.

In the ESCC patients with neoadjuvant therapy, we compared the Becker system and Japanese system for assessment of VRTC, and tried to explore the prognostic factors.

## Methods

### Patients

This study was based on a retrospective review of 175 patients who underwent surgical resection following 2 to 3 cycles of neoadjuvant chemotherapy (nCT) or neoadjuvant chemoradiotherapy (nCRT) for ESCC, between November 2009 and December 2015 at Zhongshan Hospital, Fudan University, Shanghai, China. They were diagnosed as locally advanced-stage disease (clinical T3–4), using endoscopy, computed tomography (CT) of the chest and abdomen, endoscopic ultrasound, and positron emission tomography (PET), and required neoadjuvant treatment as first-line treatment prior to surgical resection. Informed consents were obtained from all patients. This study was approved by the Institutional Review Board of our hospital (B2016–135) and was performed according to the ethical principles of the Declaration of Helsinki.

The patients were followed up routinely in outpatients, every 3 months in the first year and every 6 months in the second year, followed by annual evaluations. Patients who did not go to our hospital were contacted by telephone to obtain follow-up data.

### Pathological analysis

For specimens with neoadjuvant therapy in our hospital, all the suspected area of tumors were embedded and sectioned after the macroscopic examination, and all slides were evaluated by pathologists at once. For this study, all 175 surgically resected ESCC specimens were systematically reevaluated histopathologically by two experienced gastrointestinal pathologists. The histopathological review was undertaken with the pathologists blinded to the treatment results. The original tumour area was identified by signs of tumour regression changes, such as marked fibrosis, necrosis, flattening of the mucosa, or the presence of foreign body giant cell reaction. The extent of VRTC was assessed semiquantitatively, based on the estimated percentage of cancer in relation to the total cancer area [19–21]. With the repeated observation of tumour regression changes and training in evaluating standard, a consistency of 99% was achieved by the two pathologists. So far, several TRG systems have been used to assess the pathologic response to preoperative neoadjuvant therapy. In our study, the extent of VRTC was divided into four categories according to Becker regression criteria [19] or the Japanese Classification of Esophageal Cancer [20, 21].

Other clinicopathologic characteristics were also recorded, including age, gender, tumour location, tumour grade, tumour size (measured during the pathological sampling), lymphovascular invision, perineural growth, number of positive lymph nodes (LN), and the type of neoadjuvant.

### Statistical analysis

Categorical data were analyzed using χ2 test or Fisher’s exact test. Survival curves were estimated using the Kaplan–Meier method and were compared using the log-rank test. Overall survival (OS) was defined as the period from the date of surgery until the last confirmed date of survival or the date of death. Disease specific survival (DSS) was defined as the period from the date of surgery until the date of death, because of ESCC. Disease free survival (DFS) was defined as the period from the date of surgery until the date of disease progression or the date of death, because of ESCC. The Cox proportional hazard model was used to examine the association between clinicopathological factors and survival, to identify independent prognostic factors. Hazard rates (HRs) with its 95% confidence intervals (95% CIs) were used to determine the effect of each variable on outcome. Statistical analysis was performed using the SPSS software, version 21.0 (SPSS Inc., Chicago, IL), with statistical significance being considered with *P*< 0.05 (two-sided).

## Results

### Patient and general pathological characteristics

The clinical and pathological characteristics of the 175 patients are summarized in Table 1.There were 148 male and 27 female patients with a mean age of 59.8 (range 41–73) years. Tumours were most often located in mid-esophagus (*n* = 82) compared with the upper third (*n* = 29) or the lower third (*n* = 64). The mean tumour length was 2.9 (range 0.3–9.0) cm. One hundred eight patients (61.7%) were treated with preoperative chemotherapy, whereas 67 (38.3%) patients were treated with preoperative chemoradiotherapy. No residual tumour was found in 26 patients. Vessel and nerve invasion were identified in 57 (32.6%) and 56 (32.0%) tumours, respectively. In 83 patients who had positive LN status, 51 patients (61.5%) had 1–2 positive LN, 23 patients (27.7%) had 3–6 positive LNs, and 9 patients (10.8%) had more than 6 positive LNs.

**Table 1:** Clinical and Histopathological Characteristics in 175 Patients With ESCC Treated With Neoadjuvant therapy Plus Surgical Resection

	n	Percent (%)	tumour regression grade (Becker)			tumour regression grade (Japan)		
			TRG 1a-1b	%	*P* value	Grade 2–3	%	*P* value
Age					0.168			0.231
< 60	80	45.7	24	30.0		34	42.5	
≥60	95	54.3	38	40.0		49	51.6	
Gender					0.530			0.935
Female	27	15.4	11	40.7		13	48.1	
Male	148	84.6	51	34.5		70	47.3	
Anatomic location					0.118			0.221
Upper third	29	16.6	15	51.7		18	62.0	
Middle third	82	46.9	25	30.5		37	45.1	
Lower third	64	36.6	22	34.4		28	43.8	
Size					0.004			< 0.001
< 3 cm	90	51.4	41	45.6		58	64.4	
≥3 cm	85	48.6	21	24.7		25	29.4	
Lymphovascular invasion					< 0.001			< 0.001
Negative	118	67.4	59	50.0		70	59.3	
Positive	57	32.6	3	5.3		13	22.8	
Perineural growth					< 0.001			< 0.001
Negative	119	68.0	58	48.7		73	61.3	
Positive	56	32.0	4	7.1		10	17.9	
Lymph-node metastasis					< 0.001			< 0.001
Negative	92	52.6	46	50.0		57	62.0	
Positive	83	47.4	16	19.3		26	31.3	
ypN-stage					< 0.001			< 0.001
ypN0	92	52.6	46	50.0		57	62.0	
ypN1	51	29.1	12	30.8		20	39.2	
ypN2	23	13.1	4	17.4		6	26.1	
ypN3	9	5.1	0	0		0	0	
Type of neoadjuvant					< 0.001			< 0.001
Chemotherapy	108	61.7	23	21.3		30	27.8	
Chemoradiotherapy	67	38.3	39	58.2		53	79.1	
tumour regression grade (Becker)								
TRG1a	26	14.9						
TRG1b	36	20.6						
TRG2	33	18.9						
TRG3	80	45.7						
tumour regression grade (Japan)								
G0-1a	64	36.6						
G1b	28	16.0						
G2	57	32.6						
G3	26	14.9						

### Effect of neoadjuvant therapy on ESCC

**Fig. 1:**
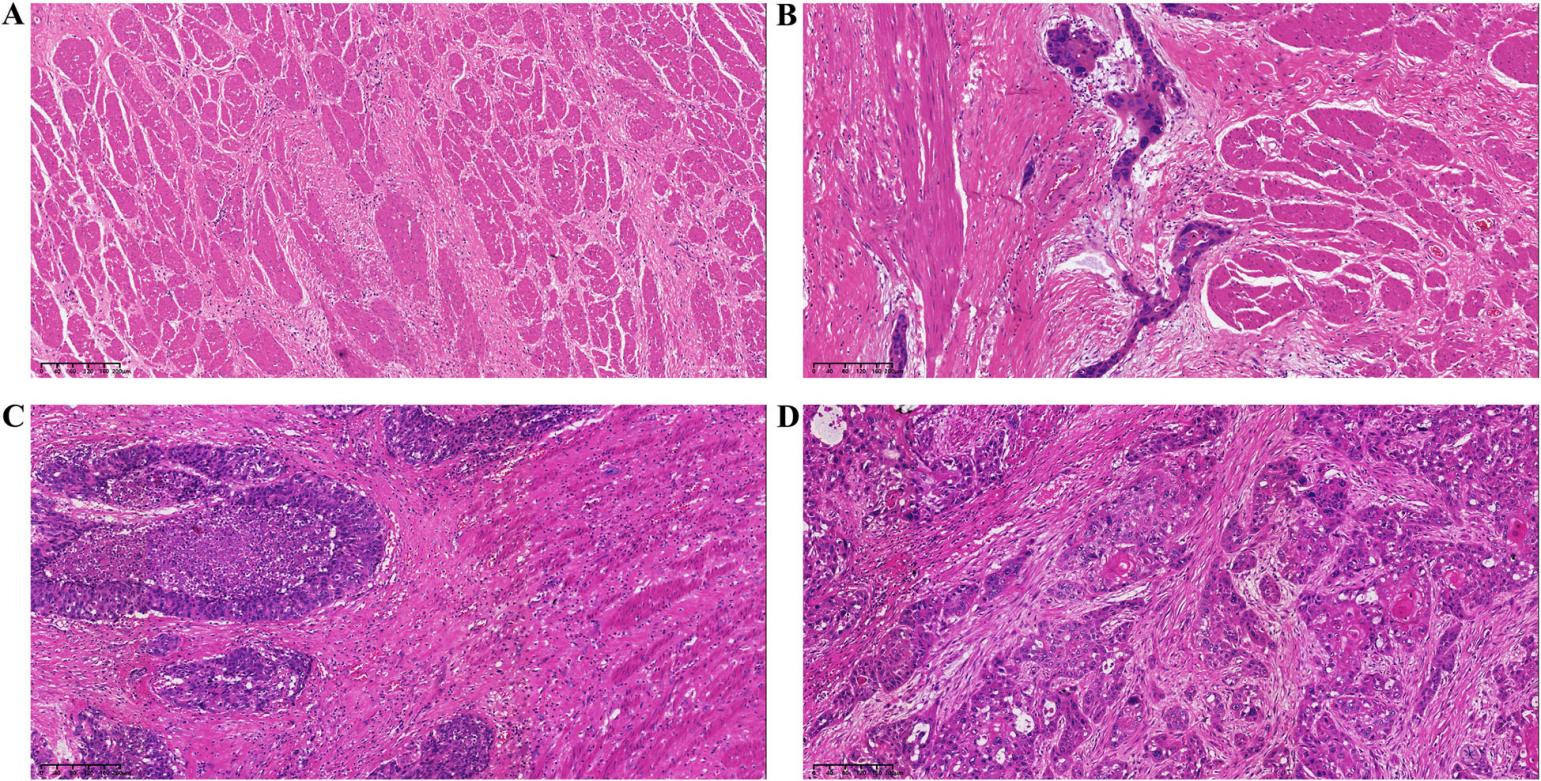
Histologic examples of different extent of visual residual tumour cells (VRTC) in ESCC treated with neoadjuvant therapy and surgery (100×): **a** no VRTC: evident fibrosis and chronic inflammatory infiltrate without detectable tumor cells (TRG 1a according to Becker system or Grade 3 according to Japanese system); **b** little VRTC: fibrosis with rare small groups of tumor cells (TRG 1b according to Becker system or Grade 2 according to Japanese system); **c** many VRTC: fibrosis and tumor cells with preponderance of tumor cells; and **d** no regression response: no signs of treatment effect

TRG system of Becker or Japanese Esophageal Cancer Association was widely accepted in western country or Japan, which was used in our study, separately. According to the Becker system, there were 26 cases (14.9%) of TRG 1a, 36 cases (20.6%) of TRG 1b, 33 cases (18.9%) of TRG 2, and 80 cases (45.7%) of TRG 3 (Fig. 1). Namely, 62 tumours (35.4%) showed a histopathological response of TRG 1a or 1b, whereas the remainder showed minor or no response (Table 1). According to the Japanese system, there were 64 cases (36.6%) of Grade 0-1a, 28 cases (16.0%) of Grade 1b, 57 cases (32.6%) of Grade 2, and 26 cases (14.9%) of Grade 3. Namely, 83 tumours (47.4%) showed a histopathological response of Grade 2 or 3, whereas the remainder showed minor or no response (Table 1).

Table 1 depicts the associations between TRG and different pathological variables. According to Becker and Japanese system, we found that more VRTC (reflecting poor response following neoadjuvant treatment) were significantly associated with longer tumour length, poorly differentiated tumour, lymphovascular invasion, perineural invasion, lymph node metastases (LNM) and single neoadjuvant treatment (Chemotherapy alone) (*P*< 0.05) (Table 1).

### Survival analysis

The median follow-up duration was 24.0 months (range, 2 to 86 months). At the time of analysis, 70 patients (40.0%) had disease progression and 59 patients (33.7%) had died of esophageal cancer. At total, 68 patients (38.9%) died. The 1-, 2-, 3-, and 5-year postoperative DFS rates for the patients in this study were 82.8, 63.9, 57.6, and 50.3%, respectively. The 1-, 2-, 3-, and 5-year postoperative DSS rates for the patients in this study were 91.0, 73.4, 60.9, and 51.0%, respectively. The 1-, 2-, 3-, and 5-year postoperative OS rates for the patients in this study were 88.8, 72.4, 56.9, and 45.6%, respectively.

### Histopathological TRG as a prognostic factor

**Fig. 2:**
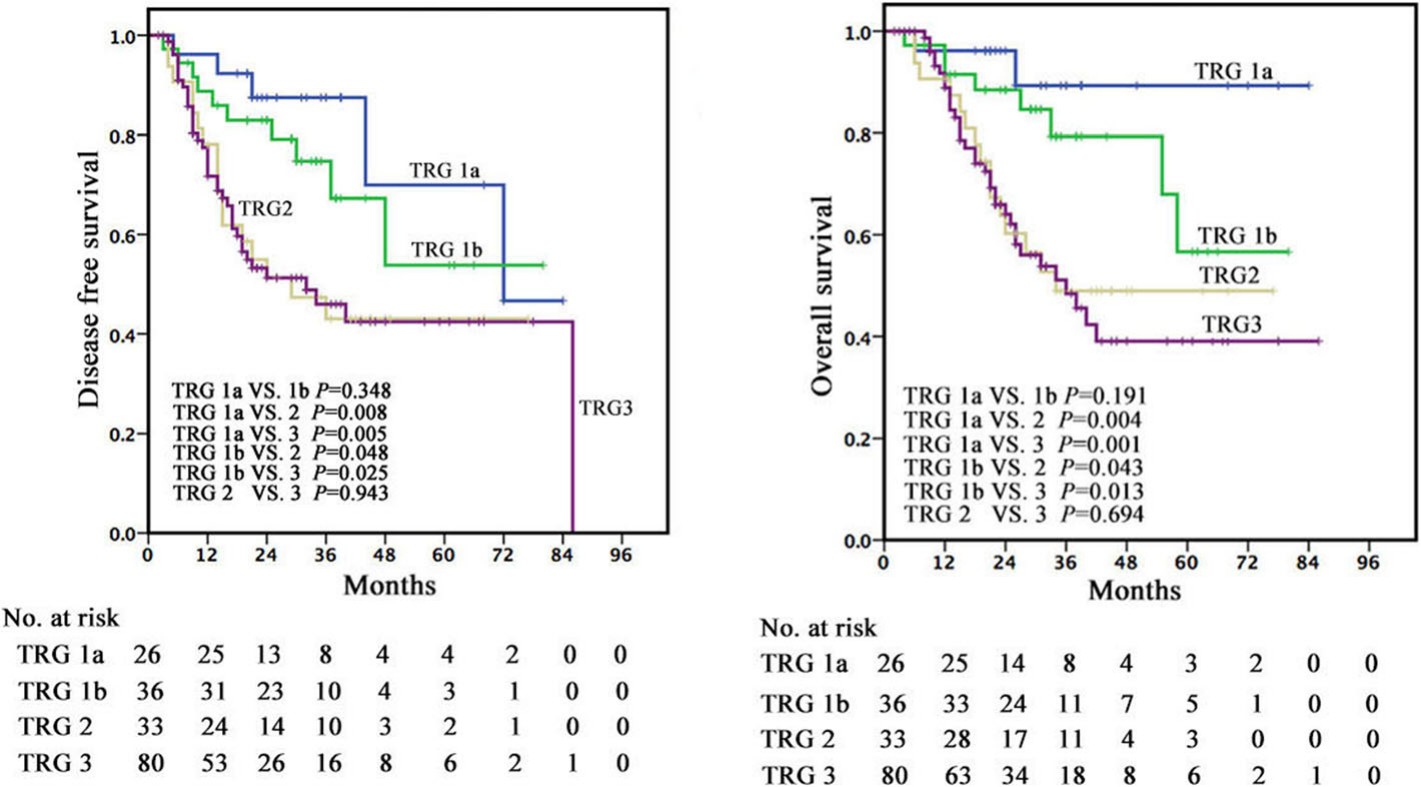
Kaplan–Meier curves for DFS and DSS in patients with ESCC treated with neoadjuvant therapy and surgery stratified by tumour regression grade according to Becker system

**Fig. 3:**
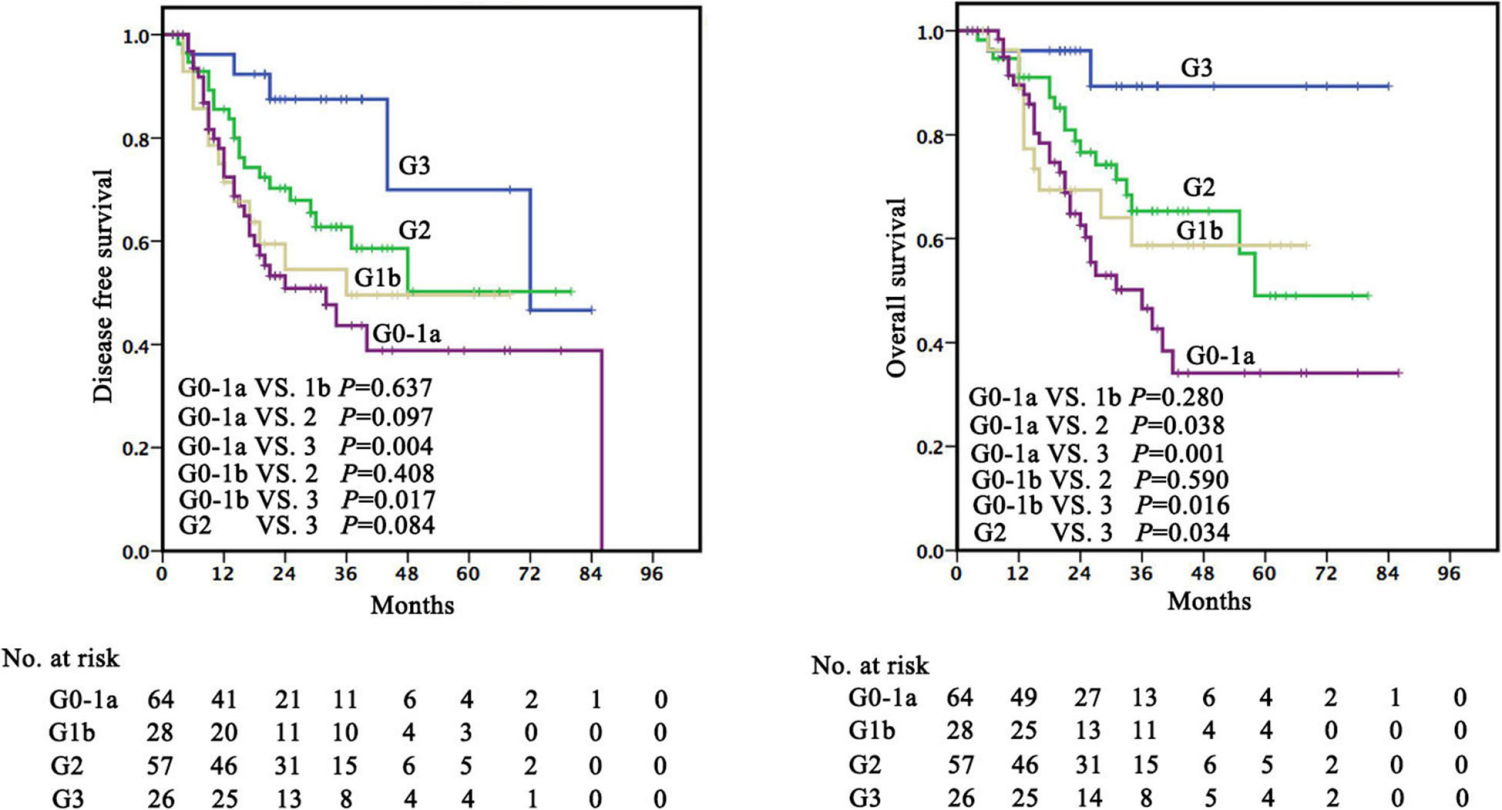
Kaplan–Meier curves for DFS and DSS in patients with ESCC treated with neoadjuvant therapy and surgery stratified by tumour regression grade according to Japanese system

Histopathological TRG was found to be strongly associated with survival. When the patients were categorized according to Becker system (TRG 1a, TRG 1b, TRG 2, and TRG 3), the 5-year DFS rates were 70.0% (median time 72 months), 53.8% (non-reached), 43.0% (29 months), and 42.4% (32 months), 5-year DSS rates were 89.3% (median time non-reached), 56.7% (median time non-reached), 49.0% (34 months), and 39.1% (36 months), and the 5-year OS rates were 72.2% (median time 78 months), 50.0% (non-reached), 50.0% (34 months), and 34.1% (34 months), respectively. Significant differences in DFS and DSS were observed between patients with TRG 1a/1b and TRG2/3 (*P*< 0.05) but not between patients with TRG1a and TRG1b (*P*> 0.05) (Fig. 2). When the patients were categorized according to Japanese system (Grade 0-1a, Grade 1b, Grade 2 and Grade 3), the 5-year DFS rates were 38.8% (median time 32 months), 49.5% (36 months), 50.2% (non-reached) and 70.0% (72 months), the 5-year DSS rates were 34.1% (median time 36 months), 58.7% (non-reached), 49.0% (58 months) and 89.3% (non-reached), and the 5-year OS rates were 31.1% (median time 31 months), 52.8% (non-reached), 45.4% (58 months) and 72.2% (78 months), respectively. Significant differences in DSS were observed between patients with Grade 3 and those with Grade 0-1a, 2 and 3 (*P*< 0.05) (Fig. 3).

### Independent prognostic factors

The univariate analysis showed a significant DFS difference in 5 factors: tumour grade, lymphovascular invasion, perineural growth, LNM, and TRG. The univariate analysis showed a significant DSS difference in 6 factors: tumour size, lymphovascular invasion, perineural growth, LNM, and TRG. The multivariate analysis identified that TRG (Becker system) (HR, 2.050; 95% CI, 1.084 to 3.878 for DFS) (HR, 2.367; 95% CI, 1.123 to 4.991 for DSS) was potentially independent factor of DFS and DSS. Beside TRG, LNM (HR, 1.770; 95% CI, 1.069 to 2.932 for DFS) (HR, 2.496; 95% CI, 1.416 to 4.402 for DSS) was also independent factor of DFS and DSS (Table 2).

**Table 2:** Univariate and multivariate analyses of factors associated with disease free surviva, disease specific survival and overall survival

	DFS		DSS		OS	
	HR (95% CI)	*P* value	HR (95% CI)	*P* value	HR (95% CI)	*P* value
**Univariate analysis**						
Age (< 60 VS.≥60)	0.892 (0.556–1.433)	0.638	0.716 (0.429–1.193)	0.2	0.666 (0.413–1.072)	0.094
Gender (Female VS. Male)	2.093 (0.956–4.582)	0.065	1.768 (0.801–3.906)	0.158	2.104 (0.960–4.613)	0.063
Anatomic location (Upper VS. Middle VS. Lower third)	1.145 (0.819–1.601)	0.429	1.224 (0.850–1.762)	0.277	1.049 (0.751–1.465)	0.778
Size (< 3 cm VS. ≥3 cm)	1.252 (0.779–2.013)	0.353	1.700 (1.012–2.856)	0.045	1.719 (1.060–2.789)	0.028
Lymphovascular invasion (Negative VS. Positive)	1.748 (1.082–2.824)	0.023	1.869 (1.119–3.121)	0.017	1.859 (1.152–2.998)	0.011
Perineural growth (Negative VS. Positive)	1.879 (1.157–3.052)	0.011	2.280 (1.363–3.814)	0.002	2.051 (1.262–3.333)	0.004
Lymph-node metastasis (Negative VS. Positive)	2.127 (1.316–3.438)	0.002	3.072 (1.787–5.281)	< 0.001	2.680 (1.633–4.398)	< 0.001
Type of neoadjuvant (Chemotherapy VS. Chemoradiotherapy)	1.208 (0.745–1.959)	0.443	1.224 (0.724–2.069)	0.45	1.379 (0.848–2.242)	0.195
Tumour regression grade- (Becker system) (TRG 1a+1b VS. TRG2+ 3)	2.596 (1.462–4.607)	0.001	3.365 (1.703–6.649)	< 0.001	2.436 (1.371–4.328)	0.002
Tumour regression grade- (Japanese systerm) (G0-1a+1b VS. G2+ 3)	1.863 (1.143–3.037)	0.013	2.155 (1.256–3.697)	0.005	1.888 (1.152–3.094)	0.012
**Multivariate analysis 1**						
Size (< 3 cm VS. ≥3 cm)	–	–	1.198 (0.697–2.059)	0.514	1.267 (0.763–2.104)	0.36
Lymphovascular invasion (Negative VS. Positive)	0.980 (0.565–1.699)	0.943	0.835 (0.462–1.510)	0.551	0.966 (0.551–1.693)	0.903
Perineural growth (Negative VS. Positive)	1.356 (0.799–2.301)	0.259	1.575 (0.900–2.758)	0.112	1.479 (0.869–2.516)	0.149
Lymph-node metastasis (Negative VS. Positive)	1.770 (1.069–2.932)	0.027	2.496 (1.416–4.402)	0.002	2.217 (1.315–3.736)	0.003
Tumour regression grade- (Becker system) (TRG 1a+1b VS. TRG2+ 3)	2.050 (1.084–3.878)	0.027	2.367 (1.123–4.991)	0.024	1.662 (0.867–3.187)	0.126
**Multivariate analysis 2**						
Size (< 3 cm VS. ≥3 cm)	–	–	1.190 (0.678–2.089)	0.545	1.275 (0.753–2.160)	0.366
Lymphovascular invasion (Negative VS. Positive)	1.096 (0.625–1.922)	0.749	0.945 (0.516–1.733)	0.856	1.059 (0.600–1.871)	0.842
Perineural growth (Negative VS. Positive)	1.431 (0.832–2.460)	0.195	1.674 (0.947–2.962)	0.076	1.546 (0.901–2.651)	0.113
Lymph-node metastasis (Negative VS. Positive)	1.826 (1.097–3.038)	0.020	2.621 (1.482–4.637)	0.001	2.282 (1.352–3.850)	0.002
Tumour regression grade- (Japanese systerm) (G0-1a+1b VS. G2+ 3)	1.378 (0.787–2.414)	0.262	1.393 (0.736–2.638)	0.308	1.198 (0.660–2.174)	0.552

## Discussion

In the present study, we retrospectively analyzed the clinicopathological data and outcomes of 175 patients with ESCC who underwent nCT or nCRT followed by surgery and elucidated the relationships between the clinicopathological characteristics. We designed the current investigation specifically focusing on patients with ESCC, which is the most common histological subtype of EC, particularly in China [2]. To the best of our knowledge, less related study has been conducted in China.

### Neoadjuvant therapy

Prior to the emergence of chemotherapy and radiotherapy, surgical resection had been the curative treatment of first choice in EC. At present, neoadjuvant therapy is used widely for patients with locally advanced EC. Evidence indicates that neoadjuvant therapy (nCT or nCRT) significantly improves survival in patients with locally advanced EC compared with surgery alone [4, 6, 7]. In our series, the 5-year survival rate was 45.6%. In our center, the 5-year survival rate was 39.7% for patients undergoing surgery alone [24]. Other results [25] showed significant 13% increase in 5-year survival for neoadjuvant therapy plus surgery as compared with surgery alone.

A reliable prognostic factor for ESCC patients undergoing neoadjuvant therapy is lacking. As preoperative neoadjuvant therapy has been used increasingly in the management of locally ESCC patients, the identification of potential prognostic parameters in these patients has recently gained momentum.

### Histopathological evaluation of tumour response

Histopathological tumour response after neoadjuvant therapy is believed to be an important objective factor and has been shown to have prognostic value in several studies [12–14, 26]. Response of the primary tumour can range from the absence of response to a total response with no VRTC. There is a consensus that the patients with pathologic complete response after neoadjuvant therapy benefit from these treatment modalities with 5-year survival rates up to 60% [25, 27, 28]. Partial tumour response is a matter of ongoing debate with controversial results from different trials [23, 29]. There are several classification systems available in the literature, with classification of responders varying from a 1 to 50% of VRTC [15, 30]. We present a brief review of the literature on classification systems used to assess the pathologic response to preoperative neoadjuvant therapy, and outline the most commonly used TRG systems, to assess if tumour regression does predict statistically significant improvement in OS and DFS.

### TRG systems of Becker

Among these systems, Becker et al. categorized TRG into four grades (TRG1a, 0%, TRG1b, 1–10%, TRG2, 11–50% and TRG3, > 50%), which reflected prognosis and survival in a more object manner and widely accepted in tumour regression evaluation [19]. A step-by-step increase in tumour regression should be paralleled by a step-by-step increase in survival, however, some authors have argued whether the prognosis of cases showing 1–10% VRTC is inferior than those of patients with 0% VRTC [15, 31–33].

We reported a 5-year DFS rates of 70.0%, a DSS rate of 89.3% and an OS rate of 72.2% in TRG1a, a 5-year DFS rates of 53.8%, a DSS rate of 56.7% and an OS rate of 50.0% in TRG1b, a 5-year DFS rates of 43.0%, a DSS rate of 49.0% and an OS rate of 50.0% in TRG2, and a 5-year DFS rates of 42.4%, a DSS rate of 39.1% and an OS rate of 34.1% in TRG3. TRG1a or TRG1b have been associated with a statistically significant survival benefit compared with other regression classes. However, no statistically significant difference could be detected between TRG1a (0% VRTC) and TRG1b (1–10% VRTC) in our patients. Consistent with our results, recently published data indicated that no statistically significant difference could be detected between pathologic complete remission and microscopic residual disease in patients with EC, and TRG 1a-1b showed a statistically significant survival benefit compared with other regression classes [31, 34, 35]. This has also been reported for patients with non-small cell lung cancer [36], rectal cancer [37], as well as locally advanced gastric cancer [19] treated with neoadjuvant therapy. In our study, multivariate analysis confirmed that TRG according to Becker system was a potentially independent prognostic factor.

### TRG system of the Japanese classification of esophageal cancer

The TRG system of the Japanese Classification of Esophageal Cancer was widely used in Japanese ESCC specimens [23, 30, 38]. Given the same histological subtype in China, Japanese grading system was also evaluated in our study: G0-1a, more than 2/3 residual carcinoma, G1b, 1/3 to 2/3 residual carcinoma, G2, < 1/3 residual carcinoma and G3, 0% residual carcinoma [20, 21]. Our date demonstrated that OS was best for patients with G3 and worst for patients with G0-1a, but there was no statistical difference in survival between patients with G1b and G2, who were in an intermediate prognostic category. The system was not significantly associated with DFS and OS in our multivariate analysis.

### Comparison between Becker and Japanese system

The main difference between Becker system and Japanese system was the classification of patients with minimal residual tumour (1–10% VRTC). Becker system divided these patients into TRG 1b, as an independent group, who had the similar 5-year survival rates as the whole cohort. Japanese system incorporated these patients into G2 (< 1/3 VRTC) [19–21]. Studies using clinical response classifications according to Japanese grading system might underestimate the number of patients (1–10% VRTC) with an impressive survival benefit. However, these studies distinguished the group with partial response (< 1/3 VRTC) and the group with less response (> 2/3 VRTC), which recognized more patients with a survival benefit [23]. With the comparison of the two systems, we believed that the percentage of VRTC could also be used in regular pathology report, which would make the further study understandable and comparable.

## Conclusion

In this study, we concluded that TRG according to both Becker system and Japanese system had its advantage in risk stratification of ESCC patients undergoing neoadjuvant therapy plus surgery. Based on Becker system, TRG was potentially independent predictors of patient outcome, which was not found based on Japanese system. The classification of cases showing 1–10% VRTC made the grouping different. We would like to recommend not only TRG according to Becker’s, Japanese and other systems but also the percentage of VRTC should be reported in the pathology evaluation, which could make study results more comparable among different research groups, and more understandable for oncologists in the clinical practice. Our findings might represent a valuable addition to the current literature in light of the increasing histopathologic response evaluation of neoadjuvant therapy in ESCC.

## Data Availability

The datasets used and/or analysed during the current study are available from the cor-responding author on reasonable request.

## Abbreviations

VRTC: Visual Residual Tumour Cells
TRG: Tumour regression grading
ESCC: Esophageal squamous cell carcinoma
LNM: Lymph node metastases
EC: Esophageal cancer
nCT: Neoadjuvant chemotherapy
nCRT: Neoadjuvant chemoradiotherapy
DFS: Disease free survival DSS Disease specific survival
OS: Overall survival
CT: Computed tomography
PET: Positron emission tomography
LN: Lymph nodes
HRs: Hazard rates
